# Increased Risk of Preeclampsia in Women With a Genetic Predisposition to Elevated Blood Pressure

**DOI:** 10.1161/HYPERTENSIONAHA.122.18996

**Published:** 2022-07-07

**Authors:** Anna Kivioja, Elli Toivonen, Jaakko Tyrmi, Sanni Ruotsalainen, Samuli Ripatti, Heini Huhtala, Tiina Jääskeläinen, Seppo Heinonen, Eero Kajantie, Juha Kere, Katja Kivinen, Anneli Pouta, Tanja Saarela, Hannele Laivuori

**Affiliations:** Department of Obstetrics and Gynecology, Tampere University Hospital, Finland (A.K., E.T., H.L.).; Center for Child, Adolescent, and Maternal Health, Faculty of Medicine and Health Technology (A.K., E.T., J.T., H.L.), Tampere University, Finland.; Faculty of Social Sciences (H.H.), Tampere University, Finland.; Computational Medicine, Faculty of Medicine (J.T.), University of Oulu, Finland.; Center for Life Course Health Research, Faculty of Medicine (J.T.), University of Oulu, Finland.; Biocenter Oulu (J.T.), University of Oulu, Finland.; PEDEGO Research Unit, Medical Research Center Oulu, Oulu University Hospital (E.K., A.P.), University of Oulu, Finland.; Institute for Molecular Medicine Finland, Helsinki Institute of Life Science (S. Ruotsalainen, S. Ripatti, K.K., H.L.), University of Helsinki, Finland.; Department of Public Health, Clinicum, Faculty of Medicine (S. Ripatti), University of Helsinki, Finland.; Department of Food and Nutrition (T.J.), University of Helsinki, Finland.; Folkhälsan Research Center and Stem Cells and Metabolism Research Program (J.K.), University of Helsinki, Finland.; Broad Institute of MIT and Harvard, Cambridge, MA (S. Ripatti).; Medical and Clinical Genetics (T.J., H.L.), University of Helsinki and Helsinki University Hospital, Finland.; Obsterics and Gynaecology (S.H.), University of Helsinki and Helsinki University Hospital, Finland.; Children’s Hospital (E.K.), University of Helsinki and Helsinki University Hospital, Finland.; Public Health Promotion Unit (E.K.), University of Helsinki and Helsinki University Hospital, Finland.; Department of Government Services (A.P.), National Institute for Health and Welfare, Helsinki, Finland.; Department of Clinical and Molecular Medicine, Norwegian University of Health and Technology, Trondheim, Norway (E.K.).; Department of Biosciences and Nutrition, Karolinska Institutet, Huddinge, Sweden (J.K.).; Department of Clinical Genetics, Kuopio University Hospital, Finland (T.S.).

**Keywords:** blood pressure, hypertension, preeclampsia, pregnancy, pregnancy complications

## Abstract

**Background:**

Preeclampsia causes significant maternal and perinatal morbidity. Genetic factors seem to affect the onset of the disease. We aimed to investigate whether the polygenic risk score for blood pressure (BP; BP-PRS) is associated with preeclampsia, its subtypes, and BP values during pregnancy.

**Methods:**

The analyses were performed in the FINNPEC study (Finnish Genetics of Pre-Eclampsia Consortium) cohort of 1514 preeclamptic and 983 control women. In a case-control setting, the data were divided into percentiles to compare women with high BP-PRS (HBP-PRS; >95th percentile) or low BP-PRS (≤5th percentile) to others. Furthermore, to evaluate the effect of BP-PRS on BP, we studied 3 cohorts: women with preeclampsia, hypertensive controls, and normotensive controls.

**Results:**

BP values were higher in women with HBP-PRS throughout the pregnancy. Preeclampsia was more common in women with HBP-PRS compared with others (71.8% and 60.1%, respectively; *P*=0.009), and women with low BP-PRS presented with preeclampsia less frequently than others (44.8% and 61.5%, respectively; *P*<0.001). HBP-PRS was associated with an increased risk for preeclampsia (odds ratio, 1.7 [95% CI, 1.1–2.5]). Furthermore, women with HBP-PRS presented with recurrent preeclampsia and preeclampsia with severe features more often.

**Conclusions:**

Our results suggest that HBP-PRS is associated with an increased risk of preeclampsia, recurrent preeclampsia, and preeclampsia with severe features. Furthermore, women with HBP-PRS present higher BP values during pregnancy. The results strengthen the evidence pointing toward the role of genetic variants associated with BP regulation in the etiology of preeclampsia, especially its more severe forms.

**Novelty and Relevance**

**What Is New?**

- This study is among the first to investigate the role of polygenic risk scores (PRSs) in the onset of preeclampsia (PE).

**What Is Relevant?**

- Our results show the association between PRS for for blood pressure (BP) and gestational BP values and highlight the increased risk for PE in women with the highest BP-PRS. Our study is, to our knowledge, the first to note the association between certain PE subtypes (PE with severe features and recurrent PE) and BP-PRS.

**Clinical/Pathophysiological Implications?**

Women whose genetics predispose them to higher BP values are also at higher risk for PE and its more severe forms. The use of BP-PRS as a predictive tool is a target for further research.

Preeclampsia affects 2% to 8% of pregnancies in developed countries and is defined by new-onset hypertension and proteinuria after 20 weeks of gestation, or, in the absence of proteinuria, impaired organ function or subjective symptoms of preeclampsia.^1^ Risks of stillbirth, preterm birth, and intrauterine growth restriction increase in hypertensive disorders of pregnancy.^2–4^ Preeclampsia is a systemic vascular disorder involving endothelial dysfunction,^5^ oxidative stress, and immunologic intolerance.^6^ Hypertensive disorders of pregnancy are risk factors for maternal and offspring cardiovascular morbidity.^2,3^ With efficient predicting methods, early diagnosis and in some cases even disease prevention could be possible, leading to improved maternal and neonatal outcome.^7^

Preeclampsia has multiple subtypes with different etiologies. Early-onset preeclampsia manifests before 34+0 weeks of gestation.^8^ Preeclampsia is considered to be associated with severe symptoms if significantly increased blood pressure (BP) level (≥160/110 mm Hg) is combined with severe headache, visual disturbances, impaired liver function, renal insufficiency, pulmonary edema, or thrombocytopenia.^1^ Early-onset preeclampsia,^9,10^ preeclampsia with severe features,^11^ as well as recurrent preeclampsia^12–14^ are strongly related to chronic hypertension and future cardiovascular disease (CVD) risk.

The genetics of underlying preeclampsia is incompletely understood. However, a family history of preeclampsia increases the risk, suggesting a genetic background. In a systematic review,^15^ preeclampsia in the family was shown to increase the risk of preeclampsia 3-fold. In a recent study by the InterPregGen consortium, the maternal single-nucleotide polymorphism heritability of preeclampsia on the liability scale in Europeans was shown to be 38.1%, and certain maternal DNA variants were identified as risk factors for preeclampsia.^16^ Variants of these genes have previously been associated with BP^17^ and body mass index.^18,19^ The consortium previously reported the first genetic variants in fetal genomes predisposing mothers to preeclampsia.^20^ In addition, epigenetic changes have been suggested to account for the onset of preeclampsia.^21,22^

A polygenic risk score (PRS) demonstrates an individual’s genetic risk of a disease affected by multiple genetic variants. A PRS is formed as a weighted sum of the risk alleles found in genome-wide association studies to be associated with the disease.^23^ PRSs are an extended method as opposed to traditional genetic risk scores in which only genome-wide significant associations are taken into account in calculating the score.^24^ In a recent study, the PRS for hypertension was shown to be associated with preeclampsia.^16^ This suggests that women genetically susceptible to hypertension might be at a higher risk for preeclampsia.

This study had 2 aims. First, we evaluated the impact of PRS for BP (BP-PRS) on the risks of preeclampsia, its subtypes, and other hypertensive disorders of pregnancy in a case-control setting. Another aim of the study was to assess the effect of BP-PRS on BP during pregnancy.

## Methods

### Population

Because of the sensitive nature of the data collected for this study, requests to access the data set from qualified researchers may be sent to H.L. at the Tampere University. Data requests may require further review by national register authorities and ethical committees. A.K. had access to all data.

Nulliparous and multiparous women with a singleton pregnancy were recruited at 5 university hospitals in Finland during 2008 to 2011 in the FINNPEC study (Finnish Genetics of Pre-Eclampsia Consortium). A detailed description of the FINNPEC cohort has been published elsewhere.^25^ Our study included those women whose genetic data were available. After the cohort description was published, additional women were included since their data were processed in accordance with the FINNPEC study protocol. Our study, therefore, includes more women than reported in the original cohort description, a total of 1514 women in the preeclampsia group and 983 women as controls. All participants provided written informed consent, and the FINNPEC study protocol was approved by the coordinating Ethics Committee of the Hospital District of Helsinki and Uusimaa (149/EO/2007).

Preeclampsia was defined as hypertension (systolic BP ≥140 mm Hg or diastolic BP ≥90 mm Hg) and proteinuria occurring after 20+0 weeks of gestation. Proteinuria was defined as urinary excretion of ≥0.3 g protein in a 24-hour specimen or 0.3 g/L or two ≥1+ readings on a dipstick in a random urine determination in the absence of urinary tract infection. Preeclampsia was classified as early onset if diagnosed before 34+0 weeks of gestation.^8^ Preeclampsia with severe features was diagnosed with either markedly increased BP (≥160/110 mm Hg) or severe proteinuria (≥5 g/24 hours) combined with subjective symptoms and, preferably, objective findings referring to a severe disease form.^8^

The FINNPEC cohort includes data on participating women and their pregnancies, including detailed data on BP and maternal biological samples. BP information was obtained from maternity cards or hospital records. Measurements were performed by medical professionals.

### Genotyping and Imputation

FINNPEC samples have been genotyped using the Infinium Global Screening Array-24 v2.0 BeadChip (Illumina, Inc, San Diego, CA) at the Institute for Molecular Medicine Finland FIMM Technology Centre (University of Helsinki, Finland). Preimputation quality control has been performed with Plink, versions 1.07 and 1.9.^26^ The genome option of Plink was used to test for unexpected genetic relationships in duplicated samples, triads, and dyads. Samples with unresolved sex mismatch, a missingness rate >5%, or heterozygosity rate ±4 SDs were omitted. Also samples with non-Finnish ancestry based on MDS analysis were excluded. Variants with a missing call rate >2%, Hardy-Weinberg equilibrium *P*<1×10^−6^, or minor allele count <3 were removed. The genotyped samples were then prephased using the Eagle 2.3.5 software^27^ and imputed with Beagle, version 4.1,^28^ using a population-specific reference panel SiSu v3 imputation reference panel, which consisted of 3775 whole-genome sequenced individuals of Finnish ancestry.

### Polygenic Risk Scores

We built a BP-PRS with the PRS-CS software,^29^ which recalculates single-nucleotide polymorphism weights from genome-wide association study summary statistics and a linkage disequilibrium reference panel by utilizing a Bayesian regression framework and placing continuous shrinkage priors on single-nucleotide polymorphism effect sizes. In the PRS-CS pipeline, default parameters and a European linkage disequilibrium reference panel with 1.1 million variants derived from samples from the 1000 Genomes Project^30^ were used. Input weights came from the publicly available genome-wide association study for SBP.^17^ A total of 1 073 588 genetic variants common for the FINNPEC cohort and the linkage disequilibrium reference panel were included in the PRSs.

### Study Groups and Settings

To assess the effect of high BP-PRS (HBP-PRS) or low BP-PRS (LBP-PRS) on the risk of preeclampsia and hypertensive disorders in a case-control setting, the participants were divided into percentiles based on their BP-PRS: women with HBP-PRS (>95th percentile) were compared with those with LBP-PRS, and women with LBP-PRS (≤5th percentile) were compared with those with HBP-PRS. PRS was categorized into HBP-PRS and LBP-PRS percentiles for ease of interpretation.^31^

To evaluate BP levels in cohorts, 1514 women experiencing preeclampsia were compared with 2 control groups. Women with chronic hypertension, gestational hypertension or gestational proteinuria were counted as hypertensive controls (n=219), whereas normotensive controls (n=764) presented none of these. Women with preeclampsia in a previous but not in the current pregnancy were excluded from both control groups to control for potential confounding in genetic samples. In the cohort setting, we investigated the differences in BP values in the 3 cohorts between women with HBP-PRS and others and between women with LBP-PRS and others.

In this study, we used BP measurements from the first antenatal visit and the highest BP throughout the pregnancy. Furthermore, we calculated BP change as BP measured at first antenatal visit subtracted from the highest BP during pregnancy. We calculated whether BP-PRS correlates with BP values during pregnancy.

### Statistical Analyses

Statistical analyses were performed using IBM SPSS for Windows, version 27 (IBM Corp, Armonk, NY). Women with HBP-PRS were compared with those whose BP-PRS was lower, and similarly, women with LBP-PRS were compared with those whose BP-PRS was higher. BP values were compared in a similar manner and, in addition, in 3 groups (preeclampsia women, hypertensive controls, and normotensive controls). Correlations were calculated using the Pearson method. Normally distributed quantitative data are expressed as means and SD, whereas medians and quartiles are reported for skewed distributions. Categorical data are shown in percentages. The normality of continuous variables was assessed with the Kolmogorov-Smirnov test. Student *t* test and 1-way ANOVA were used to compare means where appropriate, and for skewed distributions, the Kruskal-Wallis test was used. The χ^2^ test was used to analyze the associations between the categorical variables. Binary logistic regression was used to calculate odds ratios (ORs) with 95% CIs. Women with preeclampsia were compared with controls (dependent variable), whereas HBP-PRS and LBP-PRS were used as independent variables. A linear regression model was used to adjust results of BP value comparisons using BP-PRS for age, body mass index, and principal components. Homoscedasticity of errors was assessed by plotting the residuals.

## Results

Preeclamptic women and hypertensive controls were older and more obese compared with normotensive control women (Table 1). There were more primiparous women in the preeclampsia group compared with the 2 control groups. Furthermore, fewer women were smokers in the preeclampsia group compared with the control groups. Family history of preeclampsia was more common in preeclamptic women. Women experiencing preeclampsia delivered at earlier gestational weeks than control women. Newborns of preeclampsia and hypertensive control women were more often small for gestational age compared with newborns of normotensive control women. In addition, in the preeclamptic group, delivery before gestational week 34+0 was more common compared with the control groups.

**Table 1.**
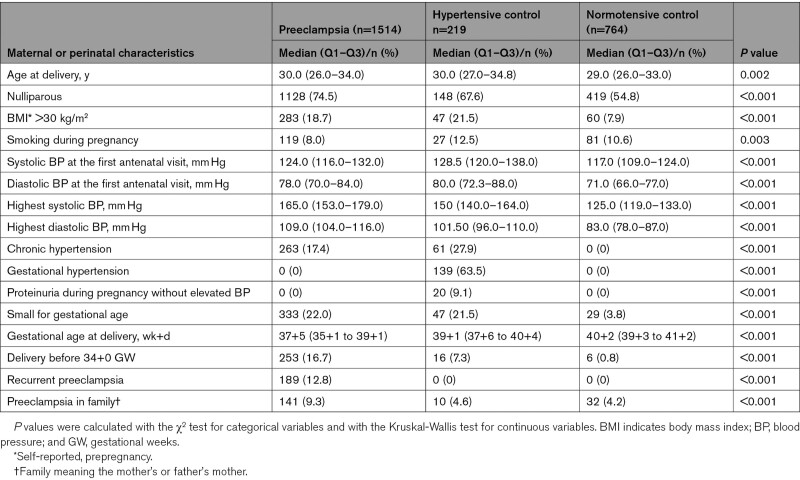
Maternal and Perinatal Characteristics

Women with HBP-PRS (>95th percentile) displayed higher BP values throughout pregnancy compared with women with LBP-PRS (Table 2).

**Table 2.**
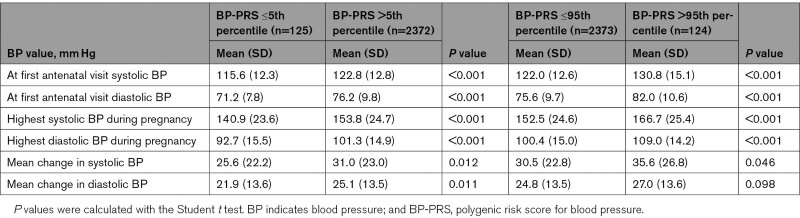
Mean BP Values Compared in Women With Low (at or Below Fifth Percentile) BP-PRS to Women With Higher BP-PRS, As Well As Women With High (Above 95th Percentile) BP-PRS to Women With Lower BP-PRS

**Figure 1.**
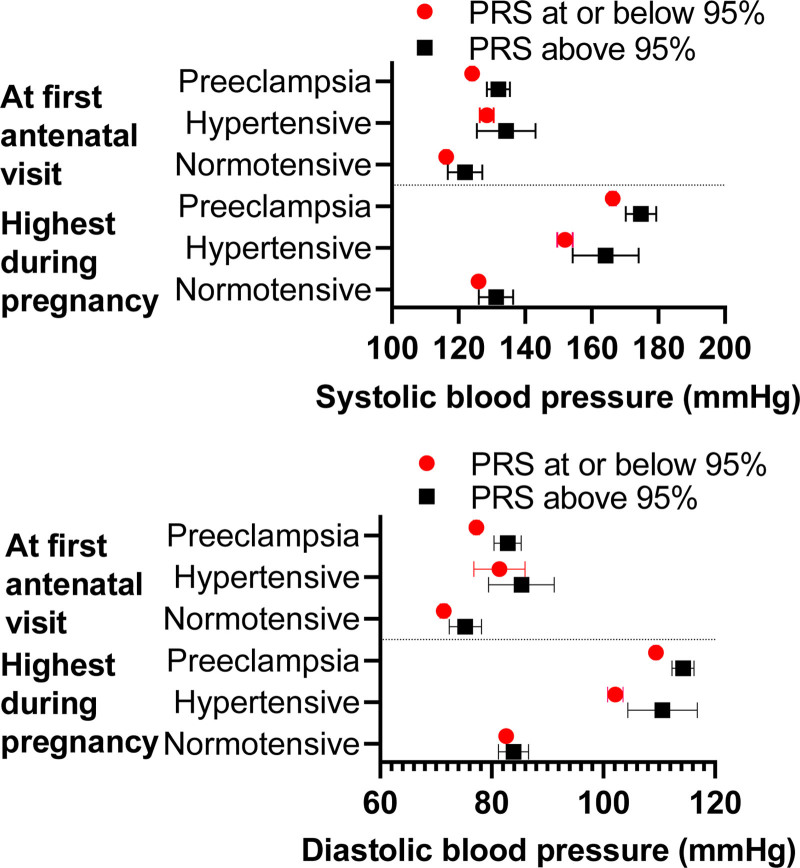
**Systolic and diastolic blood pressure (BP) values at the first antenatal visit and highest BP values during pregnancy presented in women with high (>95th percentile) polygenic risk score (PRS) for systolic BP and women with lower BP-PRS across the 3 study groups (1514 women with preeclampsia, 219 hypertensive and 764 normotensive control women).** Data are presented as means with 95% CI. Detailed data on BP values can be found in Table S1.

Among women experiencing preeclampsia, those with HBP-PRS displayed higher BP values throughout pregnancy compared with those with LBP-PRS (Table S1). The difference in BPs remained statistically significant after adjusting for age, body mass index, and genetic principal components. The increase in BP from the first antenatal visit to highest measured BP did not differ between women with HBP-PRS or LBP-PRS when analyzed within subgroups. Figure 1 shows the difference in BP between women with HBP-PRS and LBP-PRS across the 3 study groups.

Women with HBP-PRS (>95th percentile) had preeclampsia more often compared with women whose BP-PRS was lower (71.8% versus 60.1%; *P*=0.009), and women with HBP-PRS were more often hypertensive (12.9% versus 8.6%; *P*<0.001). Additionally, women with HBP-PRS were less often normotensive compared with women with LBP-PRS (15.3% versus 31.4%; *P*<0.001). In the preeclamptic group, those with HBP-PRS more often displayed preeclampsia with severe features (58.1% versus 42.4%; *P*=0.003) or recurrent preeclampsia (10.0% versus 7.7%; *P*<0.001) compared with those with LBP-PRS. Table 3 shows the proportion of women with HBP-PRS in study groups and with subtypes of preeclampsia.

**Table 3.**
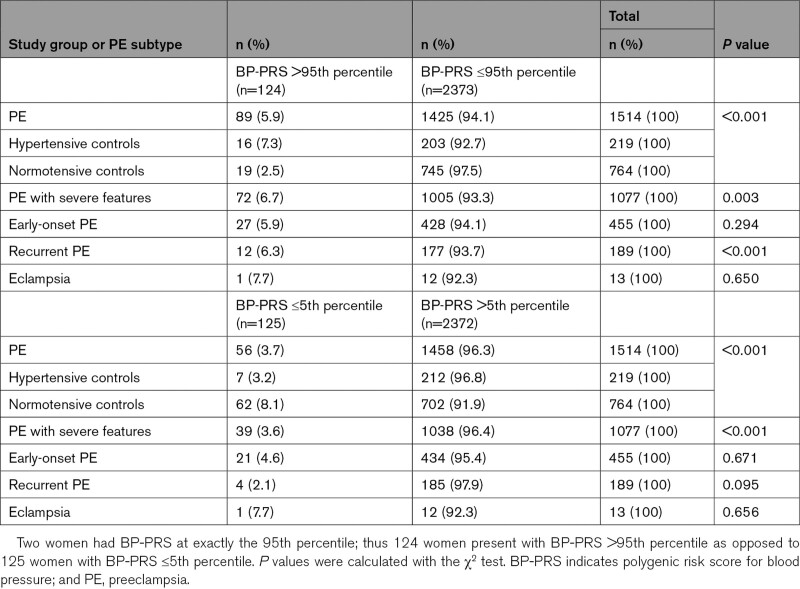
Proportions of Women With PE, Hypertensive and Normotensive Women, and PE Subtypes Compared Between Women With BP-PRS Above the 95th Percentile and Women With Lower BP-PRS and Between Women With BP-PRS at or Below 5th Percentile and Women With Higher BP-PRS

Our results demonstrate an association between HBP-PRS and preeclampsia (OR, 1.7 [95% CI, 1.1–2.5]). However, when the first antenatal BP value was included in the model, the association between HBP-PRS and preeclampsia was not statistically significant (OR, 1.33 [95% CI, 0.87–2.04]).

Women with LBP-PRS (≤5th percentile) had lower BP values throughout pregnancy compared with women with HBP-PRS. Moreover, the increase in BP from the first antenatal visit to the highest measured BP was smaller in women with LBP-PRS (Table 2).

**Figure 2.**
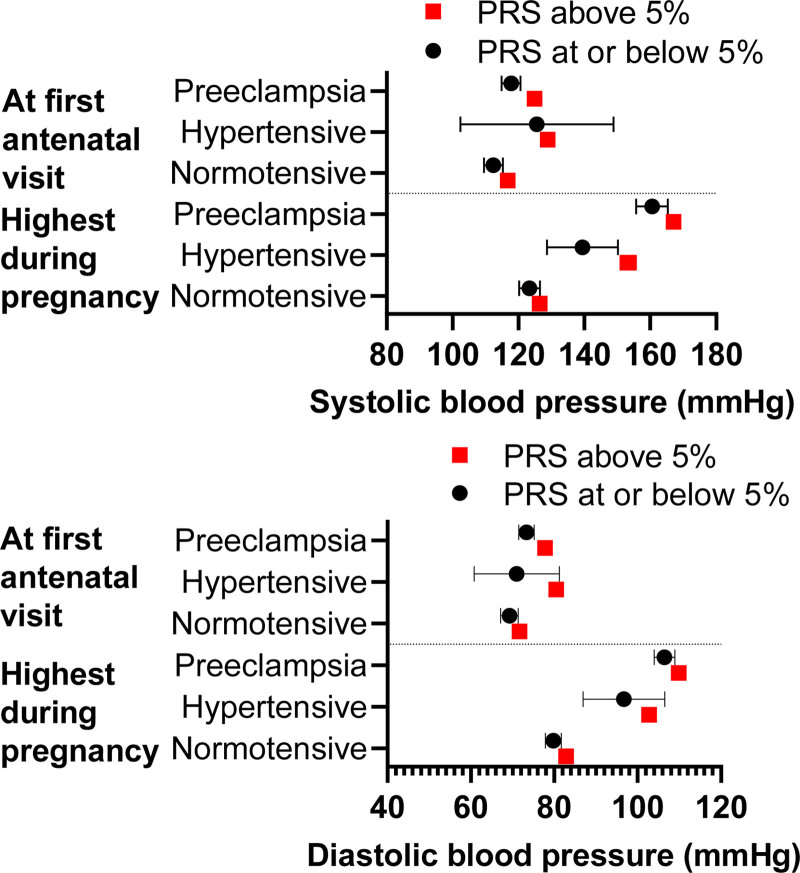
**Systolic and diastolic blood pressure (BP) values at the first antenatal visit and highest BP values during pregnancy presented in women with low (≤5th percentile) polygenic risk score (PRS) for systolic BP and women with higher BP-PRS across the 3 study groups (1514 women with preeclampsia, 219 hypertensive and 764 normotensive control women).** Data are presented as means with 95% CI. Detailed data on BP values can be found in Table S2.

In the preeclamptic group, BP values were significantly lower in women with LBP-PRS compared with others, but BP change did not show the same tendency (Table S2). The difference in BP values remained statistically significant after adjusting for age, body mass index, and genetic principal components. Figure 2 shows the difference in BP between women with LBP-PRS compared with women with HBP-PRS across the 3 study groups.

Women with LBP-PRS (≤5th percentile) had preeclampsia less frequently than those with HBP-PRS (44.8% versus 61.5%; *P*<0.001). Women with LBP-PRS had hypertensive disorders less often (5.6% versus 8.9%; *P*<0.001) and were more often normotensive (49.6% versus 29.6%; *P*<0.001) compared with those with HBP-PRS. Additionally, in the preeclampsia group, women with LBP-PRS less frequently had preeclampsia with severe features compared with those with HBP-PRS (31.2% versus 43.8%; *P*<0.001). Table 3 shows the proportion of women with LBP-PRS in study groups and with subtypes of preeclampsia.

Our results show a negative association between LBP-PRS and preeclampsia (OR, 0.51 [95% CI, 0.35–0.73]). After including the first antenatal BP value in the model, the negative association between LBP-PRS and preeclampsia remained statistically significant (OR, 0.66 [95% CI, 0.45–0.97]).

BP values from the first antenatal visit correlated with BP-PRS (correlation coefficient for SBP, 0.268; *P*<0.001). A similar correlation was seen between the highest measured BP and HBP-PRS (0.216; *P*<0.001). There was a positive correlation between BP-PRS and BP values. Correlation coefficients varied between 0.216 and 0.293 (*P*<0.001) and were convergent for both SBP and DBP values.

## Discussion

Our study of 1514 women affected by preeclampsia and 983 control women showed that preeclampsia was more common in those with HBP-PRS (BP-PRS >95th percentile) compared with others. Additionally, women with LBP-PRS (BP-PRS ≤5th percentile) were affected less frequently than others. Recurrent preeclampsia and preeclampsia with severe features were more common in women with HBP-PRS compared with others. BP values were higher in women with HBP-PRS, and the difference was observed throughout the pregnancy.

This study supports the evidence that women genetically susceptible to hypertension have an increased risk for preeclampsia in their pregnancies. In a recent meta-analysis, BP-PRS was linked to a higher risk for preeclampsia,^16^ and in previous studies, genetic variants related to hypertension have been shown to be associated with preeclampsia.^16,17^ In our study, along with the increase in preeclampsia risk, women with HBP-PRS also had higher BP values and the difference was already seen in the first antenatal visit. After adjusting for preeclampsia risk with the first antenatal BP value in women with HBP-PRS and other women, the association between HBP-PRS and preeclampsia was not statistically significant. Hence, the increase in the preeclampsia risk in women with HBP-PRS seems to be more strongly associated with prepregnancy BP levels. In our data, HBP-PRS was associated with preeclampsia with severe features and recurrent preeclampsia, supporting the evidence of genetic background in these subtypes, whereas milder forms of the disease might be linked to preexisting complex maternal conditions, such as obesity and diabetes.^6,32^ However, in our study, the incidence of early-onset preeclampsia was not associated with BP-PRS. This highlights the intricate nature of preeclampsia subtypes and the need for further research. Additionally, although LBP-PRS is associated with lower risk for preeclampsia, the background of the disease is complex and heterogenic in nature, and due to this, the use of BP-PRS as a predictive factor in these women cannot be justified.

Early-onset preeclampsia,^9,10^ preeclampsia with severe features,^11^ and recurrent preeclampsia^12–14^ are subtypes strongly related to chronic hypertension and future CVD risk.^33^ In preeclampsia, and probably these subtypes in particular, there seems to also be prepregnancy endothelial dysfunction^34,35^ influencing women’s increased risk for future CVD. In our study, HBP-PRS was associated with an increased risk for recurrent preeclampsia and preeclampsia with severe features. Thus, women with HBP-PRS might be in increased risk for a future CVD as well. In our study, the difference in BP values between women with HBP-PRS compared with others was already seen in the first antenatal visit. This might imply that these women have constantly higher BP values than those with LBP-PRS. Obviously, this might be one explanation for these women’s higher risk for CVD although preeclampsia-related vascular changes and other factors are involved. The role of BP-PRS in identifying women at higher risk for cardiometabolic complications could be a target for further investigation.

A major strength of this study is the precise BP data covering the whole pregnancy and BP measurements performed by medical professionals. In the FINNPEC data, preeclampsia diagnoses were retrospectively confirmed by a study nurse and physician to improve reliability of the diagnoses. The genetic data of the cohort are comprehensive, and investigation focusing on PRS allows for exploring large amounts of genetic information. Our case-control cohort is not matched, and only multiple pregnancies and women under the age of 18 years were excluded from the FINNPEC cohort. Consequently, our cohort represents the general obstetric population.

Additionally, a few limitations should be acknowledged. The FINNPEC cohort was recruited from the Finnish university hospitals. Due to this, the cohort might represent women with more severe symptoms or earlier disease than the general obstetric population. On the other hand, the most severe cases might not be recruited due to urgent deliveries after hospital referral. We used BP-PRS in our analyses; however, genetic variants other than BP-related ones may affect the onset of the disease. Furthermore, the hypertensive control group in our study was relatively small, impairing the power of our data. The study population was genetically mainly Finnish; thus our results cannot be directly generalized to other ethnicities. Finally, the effect of antihypertensive medications on BP could not be evaluated in our data, leading to a possible underestimation of maximum BP values and BP changes in the preeclampsia and hypertensive control groups.

## Perspectives

This study demonstrated that women with HBP-PRS are more likely to develop preeclampsia and display higher BP values during pregnancy. Additionally, LBP-PRS is associated with a decreased risk for preeclampsia. Moreover, women with HBP-PRS are affected more often by preeclampsia with severe features and recurrent preeclampsia. In clinical practice, identifying women with higher risk for preeclampsia would offer new insights for early diagnosis and more efficient management. Additionally, women with hypertensive pregnancy complications are more likely to develop CVD in the future.^10,36–38^ Identifying women with the highest risk for these complications would offer an opportunity to affect the risk factors for preeclampsia, which would also be beneficial regarding future CVD risk. The role of BP-PRS in the prediction of preeclampsia is an important target for future research. To our knowledge, few studies have investigated PRS and predisposition to hypertensive disorders of pregnancy. The results of a previous study have been convergent with our findings.^16^ Further research to confirm these results is needed. Combining maternal, paternal, and offspring PRS and their relationship to pregnancy outcomes, as well as associations between CVD-PRS and preeclampsia, provides interesting subjects for future research.

## Article Information

### Acknowledgments

We appreciate the expert technical assistance of Eija Kortelainen and late Susanna Mehtälä and contribution of the members and assisting personnel of the FINNPEC study group (Finnish Genetics of Pre-Eclampsia). In addition, we thank Prof Teemu Niiranen for his valuable comments on the manuscript.

### Sources of Funding

The FINNPEC study (Finnish Genetics of Pre-Eclampsia) was supported by the Jane and Aatos Erkko Foundation, Juho Vainio Foundation, Päivikki and Sakari Sohlberg Foundation, Academy of Finland, research funds of the University of Helsinki, government special state subsidy for the health sciences for the Hospital District of Helsinki and Uusimaa, Finska Läkaresellskapet, Liv och Hälsa Foundation, NovoNordisk Foundation, Finnish Foundation for Pediatric Research, Emil Aaltonen Foundation, Sigrid Juselius Foundation, and the Finnish Foundation for Laboratory Medicine.

### Disclosures

None.

## Supplementary Material


