# Supplementary material for: Increased Risk of Preeclampsia in Women With a Genetic Predisposition to Elevated Blood Pressure
Source: Hypertension. 2022 Jul 7;79(9):2008–15. doi: 10.1161/HYPERTENSIONAHA.122.18996 (PMC9370253; doi:10.1161/HYPERTENSIONAHA.122.18996)
Supplement: Supplementary file 1 [file hyp-79-2008-s001.pdf]

Online Supplement:

Increased risk of preeclampsia in women with a genetic predisposition to elevated blood pressure

Anna Kivioja[1,2]. Elli Toivonen[1,2]*, Jaakko Tyrmi [2-5]*, Sanni Ruotsalainen[6], Samuli Ripatti[6-8], Heini Huhtala[9], Tiina Jääskeläinen[10,11], Seppo Heinonen[12], Eero Kajantie[13-16], Juha Kere[17,18], Katja Kivinen[6], Anneli Pouta[13,19], Tanja Saarela[20], Hannele Laivuori[1,2,6,10]

1. Department of Obstetrics and Gynecology, Tampere University Hospital, Tampere, Finland
2. Center for Child, Adolescent, and Maternal Health, Faculty of Medicine and Health Technology, Tampere University, Tampere, Finland
3. Computational Medicine, Faculty of Medicine, University of Oulu, Oulu, Finland.
4. Center for Life Course Health Research, Faculty of Medicine, University of Oulu, Oulu, Finland.
5. Biocenter Oulu, University of Oulu, Oulu, Finland
6. Institute for Molecular Medicine Finland (FIMM), Helsinki Institute of Life Science, University of Helsinki, Helsinki, Finland
7. The Broad Institute of MIT and Harvard, Cambridge, Massachusetts, USA
8. Department of Public Health, Clinicum, Faculty of Medicine, University of Helsinki, Helsinki, Finland
9. Faculty of Social Sciences, Tampere University, Tampere, Finland
10. Medical and Clinical Genetics, University of Helsinki and Helsinki University Hospital, Helsinki, Finland
11. Department of Food and Nutrition, University of Helsinki, Helsinki, Finland
12. Obsterics and Gynaecology, University of Helsinki and Helsinki University Hospital, Helsinki, Finland
13. PEDEGO Research Unit, Medical Research Center Oulu, Oulu University Hospital and University of Oulu, Oulu, Finland
14. Public Health Promotion Unit, National Institute for Health and Welfare, Helsinki and Oulu, Finland
15. Children's Hospital, University of Helsinki and Helsinki University Hospital, Helsinki, Finland
16. Department of Clinical and Molecular Medicine, Norwegian University of Health and Technology, Trondheim, Norway
17. Department of Biosciences and Nutrition, Karolinska Institutet, Huddinge, Sweden
18. Folkhälsan Research Center and Stem Cells and Metabolism Research Program, University of Helsinki, Helsinki, Finland
19. Department of Government Services, National Institute for Health and Welfare, Helsinki, Finland.
20. Department of Clinical Genetics, Kuopio University Hospital, Kuopio, Finland

*These authors provided equal contribution to the study

Correspondences:
Elli Toivonen, elli.toivonen@tuni.fi
Tampere University Hospital
PL2000, 33521 Tampere, Finland

Table S1. Mean blood pressure (BP) values compared in women with high (above 95th percentile) polygenic risk score for blood pressure (BP-PRS) to women with lower BP-PRS. BP values reported within subgroups (PE, hypertensive and normotensive control groups).

BP value (mmHg)	Group	BP-PRS at or below 95th percentile n=2373		BP-PRS above 95th percentile n=124		*p*-value
		Mean	SD	Mean	SD	
At first antenatal visit systolic BP	PE	124.1	12.3	132.0	16.2	<0.001
	Hypertensive control	128.3	14.5	134.3	9.9	0.115
	Normotensive control	116.3	10.3	121.9	10.1	0.025
At first antenatal visit diastolic BP	PE	77.3	9.5	82.8	11.3	<0.001
	Hypertensive control	79.8	10.6	85.3	8.2	0.051
	Normotensive control	71.4	8.1	75.2	5.6	0.051
Highest systolic BP during pregnancy	PE	166.3	18.6	174.8	21.7	<0.001
	Hypertensive control	151.9	16.7	164.2	19.9	0.006
	Normotensive control	126.0	10.9	131.3	10.7	0.039
Highest diastolic BP during pregnancy	PE	109.4	9.2	114.3	9.5	<0.001
	Hypertensive control	102.0	11.0	109.6	11.8	0.004
	Normotensive control	82.6	6.9	83.9	5.6	0.411
Mean change in systolic BP	PE	42.2	19.6	42.8	25.7	0.842
	Hypertensive control	23.7	17.7	27.0	21.3	0.500
	Normotensive control	9.6	11.1	7.2	11.5	0.377
Mean change in diastolic BP	PE	32.2	10.4	31.1	11.9	0.363
	Hypertensive control	22.5	10.9	24.3	11.0	0.546
	Normotensive control	11.2	7.2	8.6	6.1	0.136

P-values were calculated with Student's T-test.

Table S2. Mean blood pressure (BP) values compared in women with low (at or below 5th percentile) polygenic risk score for blood pressure (BP-PRS) to women with higher BP-PRS. BP values reported within subgroups (PE, hypertensive and normotensive control groups).

PE subtype or BP value (mmHg)	Group	BP-PRS at or below 5th percentile n=125		BP-PRS above 5th percentile n=2372		*p*-value
		Mean	SD	Mean	SD	
At first antenatal visit systolic BP	PE	117.8	10.7	124.8	12.7	<0.001
	Hypertensive control	125.6	25.1	128.8	13.9	0.555
	Normotensive control	112.4	10.8	116.8	10.2	0.002
At first antenatal visit diastolic BP	PE	73.3	6.9	77.8	9.8	<0.001
	Hypertensive control	71.0	11.0	80.5	10.4	0.018
	Normotensive control	69.3	7.8	71.6	8.1	0.032
Highest systolic BP during pregnancy	PE	160.5	18.0	167.0	18.9	0.012
	Hypertensive control	139.4	11.6	153.3	17.2	0.036
	Normotensive control	123.3	12.5	126.4	10.7	0.034
Highest diastolic BP during pregnancy	PE	106.4	9.3	109.8	9.3	0.007
	Hypertensive control	96.7	10.6	102.8	10.2	0.127
	Normotensive control	79.8	7.4	82.9	6.8	<0.001
Mean change in systolic BP	PE	42.7	18.0	42.3	20.1	0.883
	Hypertensive control	13.9	17.0	24.3	17.9	0.130
	Normotensive control	10.7	12.9	9.5	10.9	0.445
Mean change in diastolic BP	PE	33.1	9.2	32.1	10.6	0.488
	Hypertensive control	25.7	6.6	22.5	11.0	0.447
	Normotensive control	10.6	7.0	11.2	7.2	0.545

P-values were calculated with Student's T-test.
